# Arrhythmias in Patients with Pulmonary Hypertension and Right Ventricular Failure: Importance of Rhythm Control Strategies

**DOI:** 10.3390/jcm13071866

**Published:** 2024-03-24

**Authors:** Suneesh Anand, Edmond M. Cronin

**Affiliations:** Section of Cardiology, Department of Medicine, Lewis Katz School of Medicine at Temple University, Philadelphia, PA 19140, USA; suneesh.anand@tuhs.temple.edu

**Keywords:** arrhythmias, pulmonary hypertension, atrial fibrillation, atrial flutter antiarrhythmia drugs, rhythm control catheter ablation

## Abstract

Arrhythmias frequently complicate the course of advanced pulmonary hypertension, often leading to hemodynamic compromise, functional impairment, and mortality. Given the importance of right atrial function in this physiology, the restoration and maintenance of sinus rhythm are of critical importance. In this review, we outline the pathophysiology of arrhythmias and their impact on right heart performance; describe considerations for antiarrhythmic drug selection, anesthetic and periprocedural management; and discuss the results of catheter ablation techniques in this complex and challenging patient population.

## 1. Introduction

Pulmonary hypertension (PH) is a chronic, progressive disease that arises from abnormally high pressures in the vessels between the heart and lungs. Several complications can occur in this serious disease, of which cardiac arrhythmias contribute significantly to increased morbidity and mortality [1]. In the last decade, emerging new strategies have led to improvements in an otherwise poor prognosis. The most recent guidelines published by the European Society of Cardiology (ESC) and European Respiratory Society (ERS) define PH as a mean pulmonary artery pressure of >20 mmHg at rest, measured using right heart catheterization (RHC) [2]. All types of PH carry a risk of cardiac arrhythmia [3,4]. However, PH from left heart disease (group 2) and lung disease (group 3) are distinct heterogenous entities with distinguishing pathophysiologies, and their management is largely directed toward the underlying cause. In this article, we focus on pulmonary arterial hypertension (PAH) (group 1) and chronic thromboembolic pulmonary hypertension (CTEPH) (group 4), for which a large amount of data are available. PAH can be idiopathic (IPAH), heritable (HPH), or associated with other conditions, such as connective tissue disease; portal hypertension; infections such as human immunodeficiency virus (HIV) infection and schistosomiasis; exposure to drugs such as anorexigens or methamphetamine; and congenital heart disease [2]. PAH and CTEPH are both characterized by progressive vascular remodeling and the obliteration of pulmonary vessels, resulting in an increase in pulmonary vascular resistance and chronic right ventricular pressure overload [5]. Arrhythmias in PAH are commonly supraventricular arrhythmias (SVA), of which atrial fibrillation (AF) and atrial flutter (AFL) are particularly prevalent. In this review, we outline the pathophysiology of arrhythmias in PH, the importance of the right atrial contribution to the overall right heart function, and how arrhythmias can be detrimental in these patient populations. We also discuss the current management, especially the significance of rhythm control strategies, considerations for antiarrhythmic drug and ablation strategies, and periprocedural management in this complex patient population.

## 2. Pathogenesis

Multiple elements contribute to the increased susceptibility to arrhythmias in PAH/CTEPH patients [6]. An increase in the right atrial (RA) chamber size along with elevated RA pressure, which reflect advanced PH disease, are risk factors for the development of arrhythmias [7]. In addition to these changes, the electrophysiological changes occurring in the RA chamber, along with sympathetic overdrive, contribute to the increased risk of arrhythmias in PH patients [8]. These changes may be particularly relevant to the relatively frequent development of typical right atrial flutter in patients with PH.

The increase in the RA chamber size is caused by the upstream transmission of pressures from pulmonary circulation and increased right ventricular pressure. Chronic hypoxia associated with pulmonary hypertension along with the pressure overload and stretching leads to fibrosis and local tissue heterogeneity within the RA. This contributes to the formation of an arrhythmogenic substrate in the RA [6,8].

Electrophysiological remodeling occurs due to changes in the expression and function of ion channels in cardiomyocytes [6]. Electrophysiological studies (EPS) performed with patients with longstanding idiopathic PAH have shown slower conduction with regional abnormalities such as reduced tissue voltage and regions of electrical silence, consistent with the presence of atrial fibrosis [9]. There were also increased areas of complex fractionated activity, which are critical sites for arrhythmia perpetuation.

Derangement in autonomic tone with sympathetic overdrive is another contributor to the increased risk of arrhythmias [6,8]. Patients with PAH have increased sympathetic activity [10]. The sympathetic autonomic system is recognized to play a significant role in the initiation and perpetuation of arrhythmias via enhanced automaticity, triggered activity, and an increase in delayed afterdepolarizations.

The risk of arrhythmias in PH is largely related to disease severity, as evidenced by correlations with various invasive and echo measures [1,3,11,12]. Hyperthyroidism is more common in PAH due to an association with other autoimmune conditions and is a risk factor for atrial arrhythmias [13]. Standard risk factors for atrial fibrillation, such as an advancing age, obesity, obstructive sleep apnea, and hypertension, almost certainly predispose patients with PH to the development of AF, although specific data are lacking.

## 3. Incidence

There is a scarcity of studies on the true incidence and prevalence of supraventricular arrhythmias in this cohort of patients. Retrospective studies have shown an incidence of 10–25% [4,14]; however, a major caveat is that these studies included only the short-term monitoring of “snapshots” in time using 12-lead ECGs or short-term Holter monitors. These methods significantly underestimate the true prevalence. In a prospective cohort study, 24 patients with PAH and 10 with CTEPH without previous arrhythmias were monitored through an implantable cardiac monitor for a median of 594 days [15]. Arrhythmias were seen in 38% of the patients during long-term continuous monitoring. SVTs SVA were the most common arrhythmias, with 16% of the episodes being atrial fibrillation and 84% being other types of SVAs like atrial ectopic tachycardia, atrio-ventricular re-entry tachycardia and atrioventricular nodal reentrant tachycardia (AVNRT). Additionally, three patients experienced bradycardia, including one resulting in syncope and a subsequent pacemaker implantation. None of the patients developed sustained ventricular arrhythmias. Other prospective studies using symptom-driven or opportunistic screening found an incidence of 25.1% over 5 years in a population with IPAH or CTEPH, and 15.8% in patients with IPAH [1,12]. As atrial flutter is particularly common in patients with PH, the careful analysis of 12-lead ECGs should be emphasized to distinguish between atrial fibrillation and atrial flutter, the latter of which is particularly amenable to catheter ablation (see “Catheter ablation” below).

Pulmonary hypertension complicates the course of approximately 5–10% of adult patients with congenital heart disease, and SVAs are particularly common in this group [16]. PH in patients with congenital heart disease can be classified as either group 1 or 2, and present a highly heterogenous group with variable cardiac anatomy and sometimes congenital abnormalities of the pulmonary vascular tree even leading to segmental PH, where some lung segments are affected and others are not [17]. PH can persist or even develop after the closure of a shunt. At its extreme, chronic left heart to right heart shunting along the pressure gradient leads to irreversible changes in the pulmonary vascular tree and eventually the reversal of the direction of the shunt, which is termed Eisenmenger syndrome.

Ventricular arrhythmias like ventricular tachycardia (VT) and ventricular fibrillation (VF) are relatively rare in PAH. There were no cases of sustained ventricular arrhythmia noted in 46 patient years of continuous monitoring using implantable loop recorders in 24 PAH and 10 CTEPH patients [15]. In a multi-center retrospective analysis of arrhythmias during cardiopulmonary arrest in 132 PH patients, the initial rhythm was bradycardia (not further specified) in 45% of cases, electromechanical dissociation in 37 cases (28%), asystole in 19 cases (15%), ventricular fibrillation in only 10 cases (8%), and other arrhythmias in 6 cases (4%) [18]. In a separate retrospective study on 26 PAH patients who underwent cardiopulmonary resuscitation for in-hospital cardiac arrest, the initial rhythm was VT/VF in only one, with pulseless electrical activity in the remainder [19]. In a retrospective cohort of patients with congenital heart disease and PH, only 3 of 310 patients developed sustained VT over a median follow-up of over 6 years [20]. These studies are indicative of the overall low incidence of VT/VF in PAH/CTEPH patients when compared to the much higher incidence in patients with predominantly left ventricular failure.

## 4. Significance of Atrial Arrhythmias in PH

Normal right heart function requires both the right ventricle and the right atrium. Of this, approximately 70% of the RV output is dependent on RV contraction and the remaining 30% on RA contraction. Tricuspid annular plane systolic excursion (TAPSE) measures the total displacement (from base to apex) of the tricuspid valve annulus from end-diastole to end-systole and can be divided into atrial and ventricular components. A study comparing the RA function of 31 PAH patients to a that of a control group of 35 patients without cardiovascular disease noted that RA function accounts for approximately 32% of TAPSE in normal patients, compared to 51% in patients with PAH [21]. TAPSE improved with PAH-specific therapy, but the RA still contributed approximately half of the total right heart function. This mirrors other pathologies, such as an RV infarction, where RV dysfunction is compensated for by RA function. Supraventricular arrhythmias can result in the loss of atrial function due to loss of atrial contraction (atrial fibrillation); rapid, and thus, less effective, contraction (atrial flutter and tachycardia); and loss of atrioventricular synchrony. This explains why supraventricular arrhythmias are poorly tolerated among patients with PAH and CTEPH.

A relationship between SVAs and clinical deterioration in patients with PH has been established by several studies (Table 1) [1,4,12,14,22,23,24,25,26]. In a prospective cohort follow-up of 317 PAH patients, 42 patients developed SVAs, of which 90.1% (38/42) required hospitalization because of RV failure [11]. Of those hospitalized, 36.8% (14/38) were admitted to the medical intensive care unit and 15 (39.4%) patients needed vasopressor support.

In another prospective cohort of 157 patients with PAH and 82 patients with inoperable CTEPH for 5 years, nearly all (97.5%) patients with an SVA clinically deteriorated with a worsened NYHA functional class or right heart failure [12]. Similar findings were reported from a 6-year, retrospective, single-center analysis, in which 231 consecutive patients with PAH or inoperable CTEPH were followed, and 84% of the patients with atrial arrhythmias decompensated with a worsened functional class or right heart failure [14].

All 40 of the patients out of 280 PAH patients who experienced a clinical worsening with an SVA improved after the restoration of sinus rhythm [1]. The reversal of cardiac decompensation in PAH patients is possible with the restoration of sinus rhythm. This observation is consistent with those of several previous studies [1,4,11,14]. It is pertinent to note that the PAH patients who developed permanent AF continued to significantly worsen in comparison to the patients who had transient paroxysmal episodes of AF. The five-year survival of patients with PAH or inoperable CTEPH was 68%, which was reduced to 58% if the patient developed a transient SVA and decreased even further to 47% for those with a permanent SVA [3]. Using a hazard ratio to calculate the mortality risk from two prospective studies examining SVA in PAH patients, permanent SVA was found to be associated with increased mortality (HR = 2.3–3.8) compared to transient SVA or no SVA.

In a retrospective single-center study on patients with congenital heart disease and PH (over half of whom had Eisenmenger syndrome), arrhythmia, mostly SVA and AF, was associated with symptoms in 75% of cases. Arrhythmia was a strong predictor of death, even after adjusting for other variables [20].

Together, these observations suggest that the occurrence of SVA in PAH patients may be an independent cause of clinical decline leading to increased morbidity and mortality [11]. Sinus rhythm ensures that the active, synchronous atrial loading of the ventricles occurs to maintain an adequate cardiac output in PAH patients. Hence, it is not only sinus restoration, but also the maintenance of the sinus rhythm that is crucial in the management of SVA in PAH/CTEPH patients.

## 5. Clinical Presentation

Symptomatology in PAH patients with cardiac arrhythmias can be variable. Most often, patients present with increasing shortness of breath, palpitations, and/or leg swelling [1]. Hemodynamic deterioration and resultant symptoms may be seen without overt palpitations, and such patients should not be classified as “asymptomatic”.

While the majority (around 80%) of patients do have symptoms at the onset of an SVA, up to 41% of episodes are asymptomatic, with their arrhythmia identified only through a screening ECG or ambulatory monitor [11]. Hence, it is pertinent to evaluate every case of right heart failure exacerbation for arrhythmias.

## 6. Management of Atrial Arrhythmias in PH

### 6.1. Emergency Management

Supraventricular arrhythmias in PH patients should be managed similarly to other populations. SVTs (not including AF and AFL) respond to intravenous adenosine (including some focal atrial tachycardias). Hemodynamically unstable patients should undergo cardioversion.

### 6.2. Rate-Control Drugs

Although rhythm control is preferable, rate control is an important initial step in management. Beta blockers and non-dihydropyridine calcium channel blockers (diltiazem and verapamil) are effective rate-control agents; however, they must be used with caution, as their negative inotropic effects may further impair RV systolic function and exacerbate hemodynamic decompensation. Intravenous amiodarone, which has less pronounced negative inotropy, may be used as a rate-control agent. Digoxin has a particular role, as it is a positive inotrope and improves cardiac output acutely in IPAH patients [28]. Its large volume of distribution, renal excretion, and narrow therapeutic window mean that its effect is slow in onset and may render dosing challenging.

### 6.3. Rhythm-Control Drugs

Many antiarrhythmics are not favorable due to their negative inotropy and chronotropy. The class 1c agents flecainide and propafenone display these properties and are also prescribed with a concomitant AV node blocker, such as a beta blocker, to prevent the 1:1 atrioventricular conduction of atrial flutter. Sotalol, a class 3 agent, is also a beta blocker. Amiodarone exhibits relatively little negative inotropy but is a negative chronotrope and may cause a variety of adverse effects with chronic use. In particular, pulmonary fibrosis is a concern, due to the difficulty in distinguishing this from pulmonary edema or underlying interstitial lung disease. Hyperthyroidism, already common in this population, may also be caused or exacerbated. Dronedarone similarly exhibits negative inotropy and, like amiodarone, interacts with CYP3A4 inducers, such as bosentan [29]. Dofetilide is a class 3 agent without inotropic or chronotropic effects that has been useful in our experience to maintain sinus rhythm in patients with PH. Its renal clearance and a narrow therapeutic window may be challenging, and it requires initiation in an inpatient setting as well as close follow-up with clinicians experienced in its use.

### 6.4. Device Therapy

Although the chronically pressure-overloaded right ventricle exhibits some similar changes to the failing left ventricle, such as fibrosis and dilation, ventricular arrhythmias are less prevalent in PH patients, and there is currently no role for an implantable cardioverter defibrillator for the primary prevention of sudden death in the absence of standard indications. Given the uncertain effects of cardiac resynchronization therapy with biventricular pacing in patients with left heart failure and right bundle branch block (RBBB), it is doubtful that it would be helpful in patients with PH [30]. Strategies for resynchronizing the right ventricle, through RV myocardial or conduction system pacing, are under investigation. Given the increasing use of conduction system pacing, it should be noted that selective left bundle branch pacing significantly increases the RV load, which may be problematic in patients with PH [31]. A strategy of device implantation and atrioventricular junction ablation should be undertaken only after careful consideration, as it does not restore atrial contraction or AV synchrony.

### 6.5. Catheter Ablation

**Figure 1:**
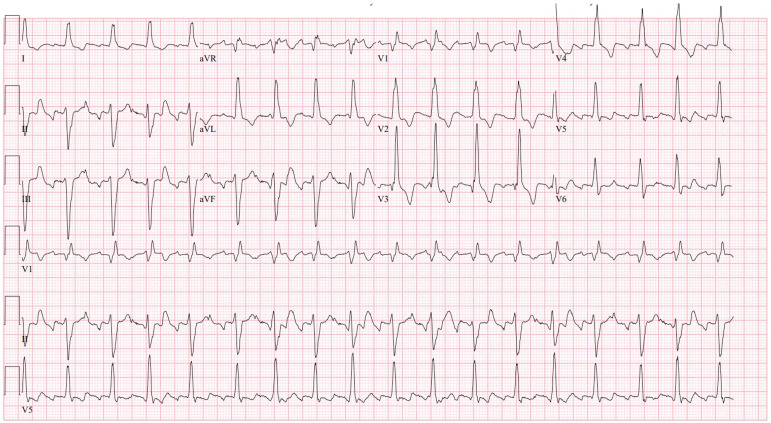
Typical atrial flutter in a patient with CTEPH with a remote pulmonary thromboembolectomy. The cycle length is 235 ms, and the right bundle branch block and left anterior fascicular block can be seen.

**Figure 2:**
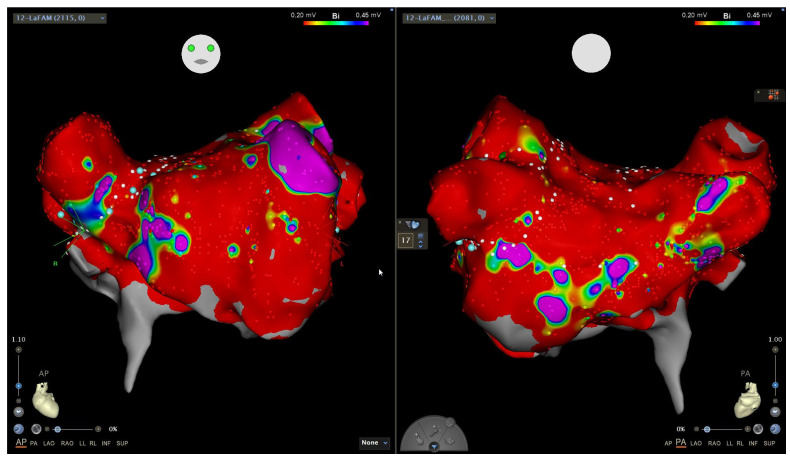
Electroanatomic voltage map of the left atrium, in atrial fibrillation, in a patient with longstanding persistent atrial fibrillation and portopulmonary hypertension. Anteroposterior (**left**) and posteroanterior (**right**) views showing a widespread dense scar (red color, bipolar voltage < 0.2 mV), with only the left atrial appendage showing normal voltage (purple color, >0.45 mV). This patient was managed with pulmonary vein isolation, left atrial appendage isolation, cavo-tricuspid isthmus ablation, and percutaneous left atrial appendage occlusion due to recurrent gastrointestinal hemorrhage, and has done well with minimal arrhythmia recurrence on low-dose antiarrhythmic drug therapy.

The safety and efficacy of catheter ablation for SVAs in the context of PH have been established by several studies, including prospective cohorts. These data stem mostly from patients with WHO PH groups 1 and 4, with typical atrial flutter being the most common arrhythmia treated with ablation. The results and safety profiles are similar to those seen in the general population, although only one study has directly compared outcomes to controls without PH [27]. Although right atrial dilation and remodeling might be expected to prolong the flutter cycle length versus controls, this has not been consistently reported [24,27]. In our experience, the flutter cycle length is not markedly different in patients with PH (Figure 1). In addition, with currently available deflectable sheaths, contact force-sensing catheters, electroanatomic mapping systems, and intracardiac echocardiography, the typical flutter ablation procedure is comparable to that in the general population. Anesthetic management (see below) often represents the most significant challenge in these cases. Ablation procedures for other right-sided arrhythmias, such as right atrial tachycardia or AVNRT, are similar to those for other conditions. Limited published data exist for left atrial ablation [32] in the setting of PH. In addition to more complex and longer procedures with additional anticoagulation considerations, transseptal puncture poses the risk of creating a persistent right-to-left shunt along the abnormal pressure gradient, leading to hypoxia. A very careful pre-procedural evaluation and medical management can ameliorate this risk, but such procedures should be carried out only in centers with considerable expertise in the management of PH. The left atrial substrate is typically more complex than that seen in patients without PH, with a more frequent presence of atypical flutters and low-voltage areas (Figure 2). Although specific data are lacking, consideration should be given to concomitant right atrial cavo-tricuspid isthmus ablation given the high prevalence of typical atrial flutter in this patient population.

#### Anticoagulation

Anticoagulation considerations are the same as for the general population with atrial arrhythmias. Many patients are already anticoagulated for other indications, such as CTEPH. Patients undergoing the cardioversion of atrial flutter and atrial fibrillation, via either pharmacologic, electrical, or catheter ablation, are recommended to be anticoagulated for 3–4 weeks prior or have left atrial appendage thrombus excluded with transesophageal echocardiography and take at least one month of uninterrupted anticoagulation afterward.

## 7. Anesthetic Management of Patients with PH

Anesthesia plays a fundamental role in several treatment strategies for arrhythmias. These include cardioversion, catheter ablation, transesophageal echocardiography, and device implantation. The continuum of sedation ranges from mild, moderate, or deep sedation to general anesthesia based on the procedure chosen.

In PH patients even low-risk procedures present an increased risk of major adverse cardiovascular events compared to the general population [33]. These increased risks include myocardial infarction, decompensated heart failure, hemodynamic instability, dysrhythmias, respiratory failure requiring prolonged mechanical ventilator support and an intensive care unit stay, and increased mortality [33].

The general approach for the perioperative management of PH patients from a sedation perspective is a multistep process focused on first determining a patient’s type(s) of PH and then individualizing the risk assessment for perioperative complications. This is followed by managing PH before procedure, especially the titration of PH-targeted therapies, and preload optimization followed by the intraoperative management of PH and postoperative ICU management, if needed [34].

### 7.1. Preoperative Risk Assessment in PH

It is imperative to involve a PH specialist in the evaluation and optimization of these patients. Preoperative risk quantification for PH group 1 can be achieved with different risk assessment tools, such as the “REVEAL 2.0” risk calculator [35] or the European Society of Cardiology (ESC)/Respiratory Society baseline risk score calculator [2]. Patients with CTEPH (group 4) are considered high-risk for noncardiac procedure; however, there are no formal risk calculators for this group.

### 7.2. Optimization of PH Prior to Procedure

The critical components of PH optimization for procedures like catheter ablation include both cardiac and pulmonary elements. The cardiac factors include preload and afterload optimization and the maintenance of coronary perfusion [36]. Diuretics are used to adjust RV preload, and inotropes are used to improve RV contractility. To reduce RV afterload, highly selective pulmonary vasodilators nitric oxide (NO) and inhaled prostanoids [34] can be used. Pulmonary considerations rely on the appropriate management of hypoxia and acidosis, which can acutely and adversely affect pulmonary vascular resistance (PVR) [36].

A complete echocardiogram is needed to evaluate for the features of PH. These include RV size and function, tricuspid valve regurgitation jet velocity, interventricular septum flattening, notching on the pulsed-wave Doppler signal of the right ventricular outflow tract, RA enlargement, and pericardial effusion [37,38].

Generally, patients are advised to continue all their PH medications up to and on the day of procedure. To maintain the NPO state, some medications such as oral prostacyclin pathway agonists may need to be substituted with parenteral or inhaled routes, since interruption in PH medications, especially the prostanoids, can result in a rebound PH crisis that is associated with increased morbidity and mortality [39]. Diuretics are also meticulously administered before procedure to achieve an euvolemic state prior to the planned procedure.

### 7.3. Intraoperative Management of PH

Once the patient is intubated and placed on mechanical ventilation, the goals are mainly to avoid hypoxia and favor mild hypocarbia (30–35 mm Hg). Ventilator settings should be modified to avoid high inspiratory pressures and positive end-expiratory pressure (PEEP). Ventilation is typically started with a tidal volume of 6 to 8 mL/kg of the ideal body weight, and a PEEP of 5 to 10 mm Hg. A PaCO_2_ of 30 to 35 mm Hg, a pH > 7.4, and an SpO_2_ > 92% are targeted [34]. A mean arterial pressure (MAP) ≥ 60 mmHg is ideal to ensure end-organ perfusion and prevent RV ischemia [40]. In addition, factors that can increase PVR, such as hypoxia, hypercarbia, acidosis, hypothermia, and pain, should be avoided [34].

### 7.4. Anesthetic Agents

The choice of anesthetic agent is dependent on the procedure and the patient. The induction of anesthesia can be associated with hemodynamic changes that can precipitate right heart failure [34]. To date, comparative studies on induction agents in patients with PH have not been carried out. Etomidate (0.15–0.3 mg/kg) has minimal effect on pulmonary artery pressure, systemic vascular resistance, heart rate, and contractility [34]. Ketamine is associated with an increase in PVR in adults and is therefore best avoided [41]. Propofol directly or indirectly adversely affects RV contractility [42] and can also cause vasodilation and may require the administration of a vasopressor or inotrope.

Caution should be taken when using benzodiazepines and opioids as premedication, since their coadministration can result in an acute increase in the PA pressures, leading to hypoxia and hypercarbia.

There is a dearth of comparative data on the effects of inhalational anesthetics on PVR, and one agent is not preferred over another [34]. If intravenous anesthesia is chosen, an infusion of propofol (50–150 µg·kg^−1^·min^−1^) can be used with caution along with opioids.

## 8. Guidelines

The 2022 ESC/ERS Guidelines for the Diagnosis and Treatment of Pulmonary Hypertension advocate for the principle of achieving and maintaining sinus rhythm in these patients as an important treatment strategy; however, these do not make specific recommendations [2]. The 2023 AHA/ACC/ACCP/HRS Guideline for the Diagnosis and Management of Atrial Fibrillation recommend a rhythm control strategy in patients with PH and AF or AFL in order to improve their functional status and perhaps improve survival [43]. Professional society guidelines specifically on the management of arrhythmias in PH are currently lacking.

## 9. Conclusions

Arrhythmias commonly complicate the clinical course of PH, frequently leading to decompensation. Convincing pathophysiologic and clinical evidence points to the restoration and maintenance of sinus rhythm as a critical goal in the management of this condition. Judicious antiarrhythmic drug selection, device therapy, cardioversion, and catheter ablation, in the context of expertise in PH management and anesthesia, can result in successful sinus rhythm maintenance with improvements in function and prognosis. Further data on the prevalence and consequences of arrhythmias are needed in groups other than PAH and CTEPH. Comparative studies on the different anti-arrhythmic drugs for patients who are not candidates for catheter ablation and on the different sedative and anesthetic agents will further the care of this complex group.

## Figures and Tables

**Table 1:** Summary of studies reporting prognostic effects of atrial arrhythmias in patients with PH and outcomes with rhythm or rate control. NR—not reported.

Study	Study Design	Study Size	Patient Population	Primary Endpoint and Results	Type of Arrhythmia	Effect of Arrhythmia	Effect of Rhythm Control
Tongers et al., Am Heart J, 2007 [14]	Retrospective, observational, single-center	231	Consecutive patients followed for PAH or inoperable CTEPH	Incidence of SVA 31 episodes of SVA were observed in 27 of 231 patients (cumulative incidence, 11.7%; annual risk, 2.8% per patient)	AFL (*n* = 15), AF (*n* = 13), and AVNRT (*n* = 3)	SVA onset was associated with clinical deterioration and right ventricular failure (84% of SVA episodes); outcome was strongly associated with the type of SVA and restoration of sinus rhythm	Mortality was 6.3% (follow-up 26 ± 23 months) when sinus rhythm was restored (all cases of AVNRT and AFL), but was 82% with sustained AF (follow-up 11 ± 8 months)
Showkathali et al., Int J Cardiol, 2011 [22]	Retrospective, observational, single-center	22	Patients with AFL and PAH or CTEPH	Success of typical atrial flutter ablation AFL ablation was acutely successful and without complications. Three patients had recurrence and underwent successful redo procedures without further recurrence	Typical atrial flutter	NR	Functional class improved in 9 and remained the same in 11 patients; 6MWT was 275 ± 141 m before and increased to 293 ± 146 m following ablation (*p* = 0.301)
Luesebrink et al., Heart Lung Circ, 2012 [27]	Retrospective, observational, single-center	38 with PAH; 196 controls	Patients undergoing ablation of cavo-tricuspid isthmus-dependent flutter with an 8 mm RF ablation catheter	Influence of PAH on typical atrial flutter ablation procedure Acutely successful ablation in all patients; patients with severe PAH had a significantly longer procedure time (78 ± 40 min vs. 62 ± 29 min; *p* = 0.033), total ablation time (20 ± 11 min vs. 15 ± 9 min; *p* = 0.02), and more ablation lesions (26 ± 16 vs. 19 ± 12; *p* = 0.018) compared to patients without PAH	Typical atrial flutter	NR	NR
Bradfield et al., JCE, 2012 [24]	Retrospective, observational, single-center	12	Consecutive patients with severe PAH (systolic pulmonary artery pressure > 60 mmHg) and AFL referred for ablation (4 congenital, 2 CTEPH, 6 PAH)	Describe flutter ablation in patients with severe PAH Acute success was obtained in 86% of procedures. Complications were seen in 14%. A total of 80% (8/10) of patients were free of AFL at 3 months; 75% (6/8) at 1 year	Typical atrial flutter	NR	SPAP decreased from 114 ± 44 mmHg to 82 ± 38 mmHg after ablation (*p* = 0.004); BNP levels were lower post ablation (787 ± 832 pg/mL vs. 522 ± 745 pg/mL, *p* = 0.02)
Kamada et al., Sci Rep, 2021 [26]	Retrospective, observational, single-center	23	13 patients with congenital heart disease; 6 with idiopathic or other PAH; 3 with CTEPH; and 1 with hemodialysis-associated PH (group 5)	Procedural success rate; short- and long-term clinical outcomes Single-procedure success, 83%; 94% (17/18) in typical atrial flutter; 73% (8/11) in atrial tachycardia (AT); and 100% (1/1) in atrioventricular nodal reentrant tachycardia.	Typical atrial flutter, atrial tachycardia, and AVNRT	NR	Antiarrhythmic drugs, serum brain natriuretic peptide levels, and number of hospitalizations significantly decreased after RFCA SVT after the last RFCA was a significant risk factor of mortality (HR, 9.31; *p* = 0.016).
Zhou et al., Front Physiol, 2021 [17]	Retrospective, observational, single-center	71	Consecutive PH patients with SVA who were scheduled to undergo catheter ablation	Feasibility and long-term outcomes of catheter ablation in PH patients with SVA Acute success in 54, complications in 4 (6.7%); during median follow-up of 36 (range, 3–108) months, 7 patients with atrial flutter experienced recurrence (78.3% success rate)	Typical atrial flutter (*n* = 33, 43.5%) was the most common SVT type, followed by atrioventricular nodal reentrant tachycardia (*n* = 16, 21.1%)	NR	NR
Cannillo et al., Am J Cardiol, 2015 [4]	Retrospective, observational, single-center	77	Consecutive patients with PAH without history of SVA	All-cause mortality and re-hospitalization During a median follow-up of 35 months, 17 patients (22%) experienced SVA. The primary endpoint occurred in 13 patients (76%) in the SVA group and in 22 patients (37%) in the group without SVA (*p* = 0.004)	Persistent AF (8 patients, 47%); permanent AF (3, 17%); paroxysmal SVA (3, 17%: 2 with atrial ectopic tachycardia and 1 with atrioventricular nodal re-entry tachycardia); right atrial flutter (2, 12%); and paroxysmal AF (1, 6%)	SVA onset was associated with the worsening of functional class, NT-proBNP, 6 min walk distance, TAPSE, and DLCO; 9 patients (53%) among those with SVA died compared with 8 (13%) among those without (*p* = 0.001)	NR
Wen et al., Am J Card, 2014 [1]	Prospective, two-center cohort study	280	Consecutivepatients > 18 years of age with IPAH at 2 national referral centers in China	All-cause mortality Patients who developed SVAs had a significantly higher mortality than those who did not; estimated survivalat 1, 3, and 6 years was 85%, 64.2%, and 52.6% vs. 92%, 81.9%, and 74.5%,respectively; *p* = 0.008	Atrial fibrillation (*n* = 16), atrial flutter (*n* = 13), and atrial tachycardia (*n* = 11)	In most patients (97.5%), the onset of SVA resulted in clinical deterioration or worsening right-sided cardiac failure	Patients who developed permanent SVA had a significantly lower survival rate than patients with transient SVA (*p* = 0.011) or without SVA (*p* < 0.001); survival was not statistically different between patients with transient SVA and those without SVA (*p* = 0.850)
Olsson et al., Int J Cardiol [12]	Prospective, single-center cohort study	239 (PAH, *n* = 157; inoperable chronic thromboembolic pulmonary hypertension, *n* = 82)	Consecutive patients ≥ 18 years of age treated for PAH or inoperable CTEPH	Incidences of AF and AFL The cumulative 5-year incidence of new-onset atrial flutter and fibrillation was 25.1% (95% confidence interval, 13.8–35.4%)	AF 50% and AFL 50%	AF and AFL were frequently accompanied by clinical worsening (80%) and right heart failure (30%); new-onset atrial flutter and AF were independent risk factors for death	Stable sinus rhythm was successfully re-established in 21/24 (88%) with atrial flutter and in 16/24 (67%) with atrial fibrillation Higher mortality was observed in patients with persistent AF compared to patients in whom sinus rhythm was restored (estimated survival at 1, 2, and 3 years was 64%, 55%, and 27% versus 97%, 80%, and 57%, respectively)
Smith et al., Pulm Circ, 2018 [25]	Retrospective, observational, multi-center	297 (group 1 PAH, *n* = 266; CTEPH, *n* = 31)	All patients in a healthcare system with PAH or CTEPH (excluding those who had undergone thromboembolectomy)	AF/AFL occurrence and survival 79 (26.5%) developed AF/AFL, either before or after a diagnosis of PH or CTEPH	AF in 46 (58.2%), atrial flutter in 25 (31.6%), and instances of both in 8 (10.1%)	AF/AFL was associated with a 3.81-fold increase in the hazard of death (95% CI, 2.64–5.52; *p* < 0.001) Mortality risk was present, whether paroxysmal or persistent AF/AFL	NR
Ruiz-Cano et al., Int J Cardiol, 2010 [23]	Retrospective, observational, single-center	282 patients with PH; not reported but implied 28 with arrhythmias	Group 1 PAH: 6 patients (26.1%) had idiopathic PAH; 7 (30.4%), a connective tissue disease; 6 (26.1%), toxic oil syndrome; and 4 (17.4%), Eisenmenger syndrome	Safety and efficacy of EPS Efficacy 100% for AVNRT and 95% for typical flutter; safety not reported	AF (*n* = 12, 42.8%); atypical flutter (*n* = 7, 25%); typical flutter (*n* = 5, 17.8%); andAVNRT (*n* = 4, 14.2%)	Most episodes of SVA (82%) were symptomatic with clinicalworsening or RV failure Clinical deterioration was not observed in patients with AVNRT	Restoration of SR was associated with a clinical improvement in all the patients, with an average increase of 196 ± 163 m in 6MWT
